# The eXtensible ontology development (XOD) principles and tool implementation to support ontology interoperability

**DOI:** 10.1186/s13326-017-0169-2

**Published:** 2018-01-12

**Authors:** Yongqun He, Zuoshuang Xiang, Jie Zheng, Yu Lin, James A. Overton, Edison Ong

**Affiliations:** 1 Unit for Laboratory Animal Medicine, Department of Microbiology and Immunology, Center for Computational Medicine and Bioinformatics, University of Michigan Medical School, Ann Arbor, MI USA; 2 Department of Genetics, University of Pennsylvania Perelman School of Medicine, Philadelphia, PA 19104 USA; 3 Center for Computational Science, University of Miami, Coral Gables, FL USA; 4 Knocean Inc., Toronto, ON Canada; 5 Department of Computational Medicine and Bioinformatics, University of Michigan Medical School, Ann Arbor, MI USA

**Keywords:** Ontology, Interoperability, eXtensible ontology development, Software, Ontoanimal tools, Ontofox, Ontobee, Ontorat, Semantic alignment, And ontology design pattern

## Abstract

Ontologies are critical to data/metadata and knowledge standardization, sharing, and analysis. With hundreds of biological and biomedical ontologies developed, it has become critical to ensure ontology interoperability and the usage of interoperable ontologies for standardized data representation and integration. The suite of web-based Ontoanimal tools (e.g., Ontofox, Ontorat, and Ontobee) support different aspects of extensible ontology development. By summarizing the common features of Ontoanimal and other similar tools, we identified and proposed an “eXtensible Ontology Development” (XOD) strategy and its associated four principles. These XOD principles reuse existing terms and semantic relations from reliable ontologies, develop and apply well-established ontology design patterns (ODPs), and involve community efforts to support new ontology development, promoting standardized and interoperable data and knowledge representation and integration. The adoption of the XOD strategy, together with robust XOD tool development, will greatly support ontology interoperability and robust ontology applications to support data to be Findable, Accessible, Interoperable and Reusable (i.e., FAIR).

## Background

In informatics, an ontology is a set of computer- and human-interpretable terms and relations that represent entities and their relations in a specific domain of the world. Hundreds of biological/ biomedical ontologies have been developed in the last two decades. The Open Biological and Biomedical Ontologies (OBO) Foundry is a collaborative initiative aimed at establishing a set of ontology development principles and incorporating ontologies following these principles in an evolving non-redundant and interoperable suite [1]. The OBO library currently includes over 160 ontologies covering >3 million terms in biological and clinical domains. NCBO BioPortal [2] has over 400 ontologies including both OBO and non-OBO ontologies. Given hundreds of ontologies developed, a critical issue is the lack of ontology interoperability, preventing the seamless understanding and exchange of semantic information between different resources.

Ontologies are widely used in different areas [3, 4], including: (1) Naming “things”; (2) Knowledge base construction, e.g., the Ontology of Vaccine Adverse Events (OVAE) representing the knowledge of adverse events induced by FDA-licensed vaccines [5]; (3) Data exchange, e.g., BioPAX for representing molecular and cellular pathways and facilitating the exchange of biological pathway data [6]; (4) Data integration, e.g., the Ontology for Biomedical Investigations (OBI) [7] for integrative representations of data in various areas of life-science and clinical investigations; (5) Data analysis, as exemplified by the wide usage of the Gene Ontology (GO) [8] to support high-throughput gene expression data analyses; (6) Natural language processing [9, 10]; (7) Metadata standard generation [11–13]. (8) Information retrieval and new knowledge discovery [14–16].

To support various needs in ontology development and applications, different software programs have been developed. The Protégé OWL editor [17] is likely the most popular tool for manual processing and editing of ontology OWL documents. However, manual ontology development is typically tedious and inefficient, especially when the structure of ontology is enormous. Over the years, we have developed a collection of web-based “Ontoanimal” tools including Ontofox [18], Ontodog [19], Ontorat [20], Ontobee [21], Ontobeep [22], Ontobull [23], Ontokiwi [24], and Ontobat [20]. Each Ontoanimal tool has its specific functions, and the collective use of these tools enables users to cover the full development of ontology and linked data (i.e., data published on the Web that it is explicitly defined, machine-readable, and interlinked with external data sets [25]), including: extracting ontology subsets for term reuse and semantic alignment, providing ontology community views, adding and editing multiple ontology terms, visualizing and comparing ontology terms, supporting community editing and discussion, and creating ontology-based linked data. The back-end He group RDF triple store serves as the default ontology RDF triple store for the OBO Foundry ontologies [21]. Complementary to Protégé, Ontoanimal tools are widely used for efficient and flexible ontology development without requiring programming skills. For example, according to Google Analytics and Google Scholar, Ontobee has been used by over 77,000 users from 181 countries, Ontofox has been used by over 17,000 users from 147 countries, and Ontoanimal tools have been cited by >400 publications in the last 5 years.

The Ontoanimal tools and other similar tools have significantly enhanced the speed and quality of ontology development and improved ontology interoperability. Given an increasing number of these tools, it would be important to identify the common features of these tools. After retrospective examination and careful summary of these tools, we realized the most common feature of these tools being their support for “extensible” ontology development. Such extensibility is crucial to increase the interoperability among the ever increasing number of ontologies. However, a systematic view of “extensible” ontology development is not available. Thus, we propose the “eXtensible Ontology Development” (XOD) strategy and four XOD principles in this paper. Such an XOD strategy is complementary to the OBO principles and the OBO goal of achieving interoperable ontology suite [1], and it is also complementary to the ten simple rules proposed for biomedical ontology development [26]. We also believe that the adoption of the XOD strategy and principles support the FAIR Guiding Principles proposal that all research data should be Findable, Accessible, Interoperable and Reusable (FAIR) for both machine and human users [27].

### XOD: eXtensible ontology development

**Fig. 1:**
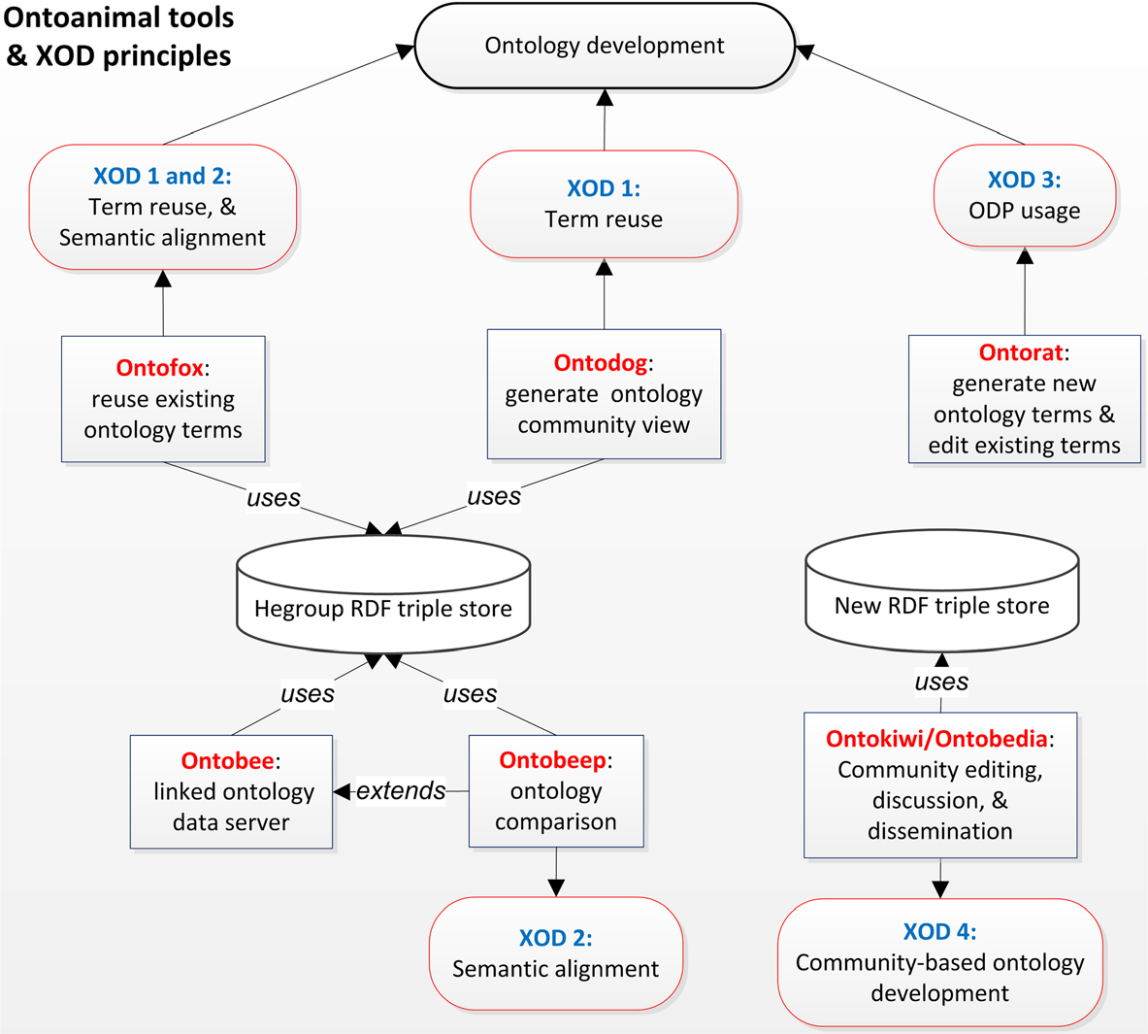
Summary of Ontoanimal tools and their features. Ontofox supports ontology reuse by extracting terms and axioms. Ontodog provides ontology community views by allowing community-preferred annotations. Ontorat automatically generates new ontology terms and edits existing terms based on ontology design patterns. Ontobee is an ontology linked data server for OBO library ontologies and many non-OBO ontologies. The Ontobee-based Ontobeep program supports ontology comparison and identification of redundant terms. Ontokiwi is a Wiki-like ontology editing and discussion program. Ontobedia is an application of Ontokiwi. Ontobat supports ontology-based data processing (e.g., conversion from Excel to OWL) and analysis. These tools support different XOD principles

In information technology, extensible describes something (e.g., a program or protocol) that is designed so that users/developers can expand or add to its capabilities with no or minimal change in the system’s internal structure and data flow. For example, extensibility is a primary feature of the eXtensible Markup Language (XML) system. Being “eXtensible”, XOD contains four key principles that are extensible at different levels of ontology development (Fig. 1):

(i) Ontology term reuse. Instead of reinventing the wheel when generating new ontologies, XOD emphasizes the reuse of terms from existing reliable ontologies that are well constructed and commonly used by the ontology community [1, 28, 29].
(ii) Ontology semantic alignment. For ontology interoperability, it is important to align imported terms from existing ontologies and newly added terms with the same semantics.
(iii) ODP usage for new term generation and existing term editing. Instead of adding one term at a time, XOD emphasizes the addition or editing of a group of terms based on ontology design patterns (ODPs).
(iv) Community extensibility. While the development of an ontology might be initiated by a small group with one or a few use cases, the ontology should be co-developed and applied to more use cases by more people in a broader community.

Ontoanimal tools (Fig. 1) and many other programs support XOD principles (Table 1). In the following sections, different principles and associated tools are described with details.

**Table 1:** XOD principles and supporting software programs

XOD principle #	XOD principle names	Tool name
XOD 1	Ontology term reuse	Ontodog, Ontofox, OntoMATON, Protégé MIREOT plugin, ROBOT
XOD 2	Semantic alignment	Ontobeep, Ontofox, ROBOT
XOD 3	ODP usage	MappingMaster, Ontorat, Populous, ROBOT, TermGenie, Webulous
XOD 4	Community extensibility	Ontodog, Ontokiwi/Ontobedia, WebProtege

### XOD 1: Ontology term reuse

Reusing terms from reliable reference ontologies is better than reinventing the wheel to generate new terms in an ontology [18, 30]. The reference ontology should be registered in an ontology library (e.g., OBO Foundry) to make these terms more findable, accessible, and reusable. To improve reusability and extensibility, ontology terms in the reference ontologies should be expressive and generalizable and endure consistency checking and evaluation. To maintain ontology interoperability, ontology term mapping is often used to map terms from different ontologies with the same meaning [31]. Compared to ontology term reuse, term mapping is less ideal since it is time-consuming, often inaccurate, redundant, and increases maintenance cost and confusion. Given multiple ontologies without using the ontology term reuse strategy, ontology mapping becomes a core task for ontology interoperability [32]. The wide usage of the term reuse principle would make the mapping among different ontologies unneeded.

An initial method of ontology term reuse was to import a full ontology, which was not ideal since it might import too many unrelated terms. Instead of importing external ontologies as a whole, the Minimum Information to Reference an External Ontology Term (MIREOT) strategy, introduced by OBI developers [30], proposes the usage of the minimal information of an external ontology term that is of direct interest to a target ontology [30]. Specifically, MIREOT suggests the following minimal set: (1) source ontology URI; (2) source term URI; and (3) target direct superclass URI. With the set of information, the source ontology term can be extracted to under the target direct superclass. Since it is often hard to maintain semantic consistency among ontologies, the popular MIREOT strategy provides a simple solution with possible semantics loss.

**Fig. 2:**
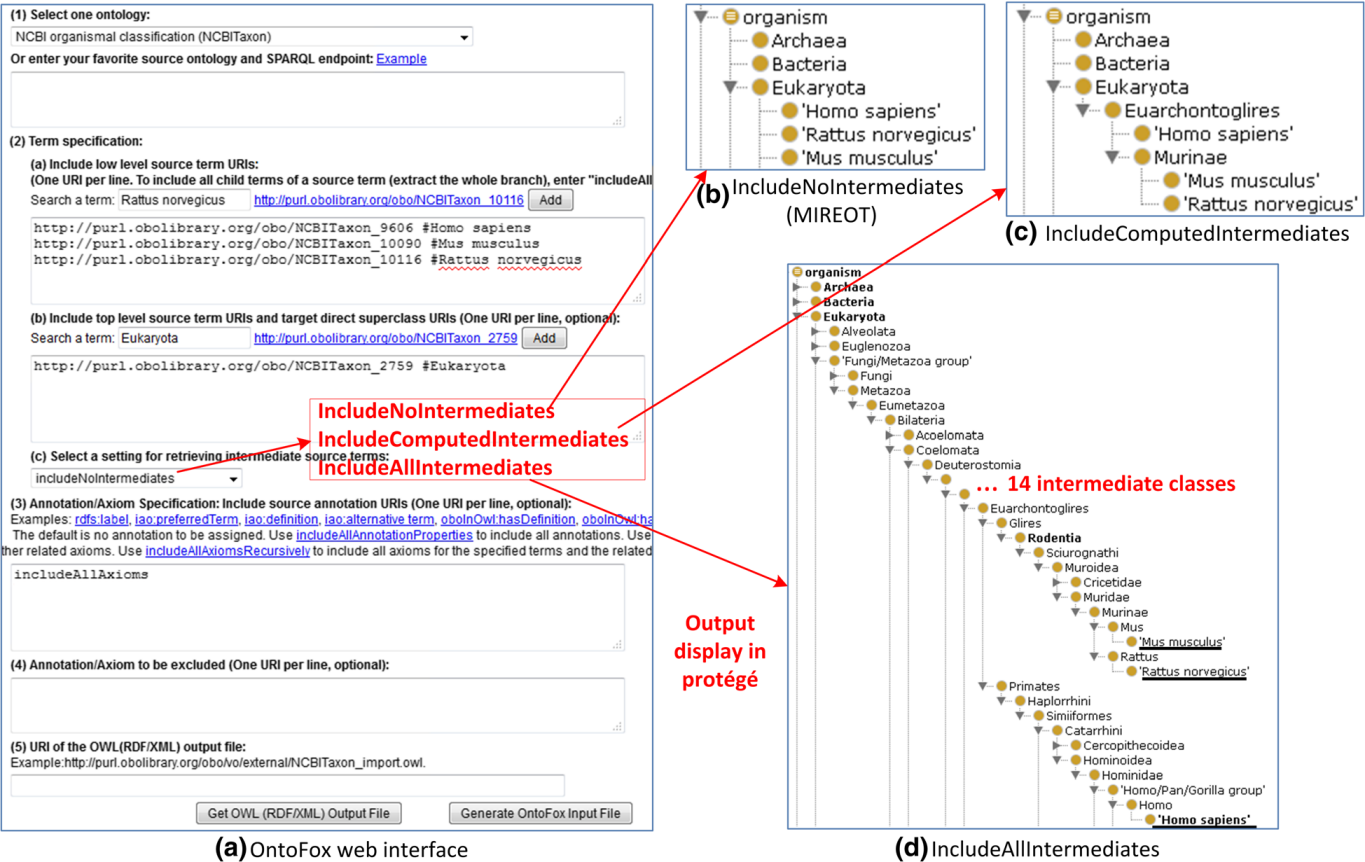
Ontofox retrieval of an NCBITaxon subset. Input data includes 3 species of organisms (human, mouse, and rat) and Ontofox settings. The input data and settings can be entered via web-based forms (**a**). The Ontofox results can be shown using Protégé (**b**-**d**). Different results may appear based on different settings: The setting “*IncludeNoIntermediates”* implements MIREOT (**b**). The setting “*includeComputedIntermediates*” extracts computed intermediates which that are closest ancestors of more than one low level source terms (**c**). The setting “*includeAllIntermediates*” outputs all possible intermediates

Ontofox, Ontodog, and Ontobull support term reuse. Originally named OntoFox, Ontofox was the first web tool to support the MIREOT strategy (Fig. 2) [18]. Ontofox is able to quickly and easily fetch user-specified terms and their annotations from source ontologies and assign them under defined superclass(es) in target ontologies (Fig. 2a and b). Ontofox also extends MIEROT by retrieving semantical axioms with different options (see next section). Ontodog is also able to extract a subset of ontology terms and axioms [19]. Unlike plain text definition in Ontofox, Ontodog uses Excel input files to identify terms to retrieve. To match possible updates of source ontologies, e.g., the upper-level Basic Formal Ontology (BFO) [33], Ontobull is developed for automatic conversion and updating [23].

Several other tools also support ontology term reuse (Table 1). The Protégé MIREOT plugin [34] and OntoMaton [35] support term reuse as a plugin of the Protégé OWL editor or Google Spreadsheets, respectively. ROBOT is a command-line Java tool supporting the extraction of ontology terms and subsets [36]. ROBOT also has many other features and supports multiple XOD principles (Table 1) as described below [36].

To better support ontology reuse and community-based ontology development, it would also be valuable to have the authors of the source ontology know of the reuse of a term in their ontology. The Ontobee program [21] includes a feature in the web page of an ontology term that shows all the other ontologies reusing the term, which supports ontology interoperability.

### XOD 2: Ontology semantic alignment

The XOD 2 principle proposes to align imported ontology terms and newly added terms with the same or compatible semantics. Such semantic alignment has two specific meanings. First, in addition to the term reuse in XOD 1, the semantic relations among reused terms should also be reused and aligned. If different relation types (i.e., object properties) mean the same thing, they should be merged. Correspondingly, the axioms of reused terms and any additional terms specified in the axioms should also be retrieved and imported. Second, the semantics related to newly developed terms should be aligned and compatible with imported semantics, and the same or compatible relations be used in the new ontology. If a well-defined relation already exists, we should reuse the relation instead of defining another relation with the same meaning. Such semantic alignments support ontology semantic interoperability.

Ontofox and Ontodog support ontology semantic alignment. Ontofox and Ontodog extract semantic axioms and terms related to user-specified terms from the source ontologies. Given different options, Ontofox allows the computation and extraction of (i) intermediate terms that are the shared parent terms of multiple low level terms (Fig. 2c), or (ii) all intermediate terms between the required terms and a top level term (Fig. 2d). These subset semantic axioms and terms can then be retrieved and become a part of the new ontology. Note that manual intervention and judgment may still be needed now to ensure the semantic alignment between retrieved subset and target ontology semantics [37]. It will also be important to have computer-supported semantic capture and synchronization of ontology evolution and updates. To foster reliability, an overall formal evaluation and consistency checking would be needed.

### XOD 3: ODP-based ontology development

An Ontology Design Pattern (ODP) represents a reusable solution to solve a recurrent modeling problem in the context of ontology engineering [20, 38, 39]. ODPs provide extensible representations of entities and relations, make ontologies more maintainable, and improve ontology quality. This XOD 3 principle requires an ODP-based strategy to develop and edit new terms, annotations, and relations. This principle extends XOD 1 and XOD 2 and provides a specific, feasible, and robust mechanism to achieve interoperable ontology term generation/annotation and semantic consistency.

**Fig. 3:**
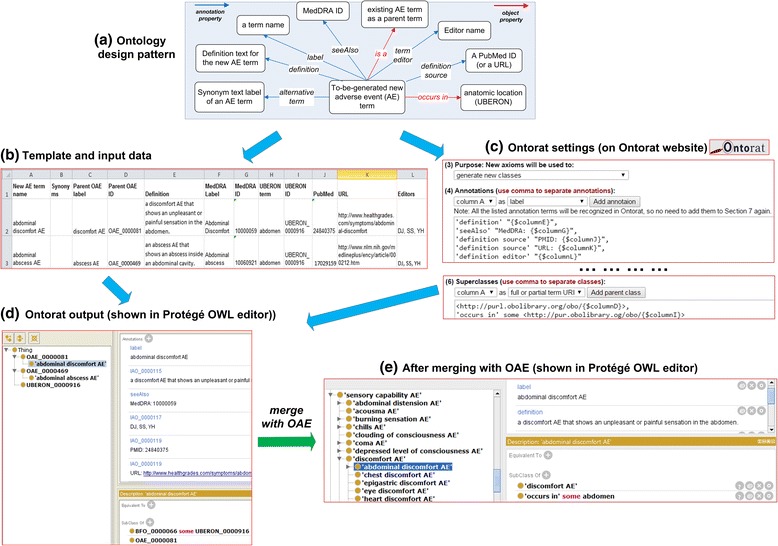
New OAE term generation and annotation using Ontorat. First an ODP was identified to define new AE terms (**a**). The ODP guided the generation of an Excel template and Ontorat settings. The template file was populated with detailed contents (one row for one new term; only two rows shown in this example) (**b**). The Ontorat settings were matched to the Excel data format (**c**). The settings and populated Excel file were then used as Ontorat inputs to generate an OWL format output file containing newly created ontology terms together with their annotations. The output could be displayed using the Protégé OWL editor (**d**). After merging the output file to existing OAE file, the detailed information of imported ontology terms (e.g., ‘discomfort AE’ OAE_000081) seen in (**d**) will be obtained from and aligned to existing OAE (**e**)

**Fig. 4:**
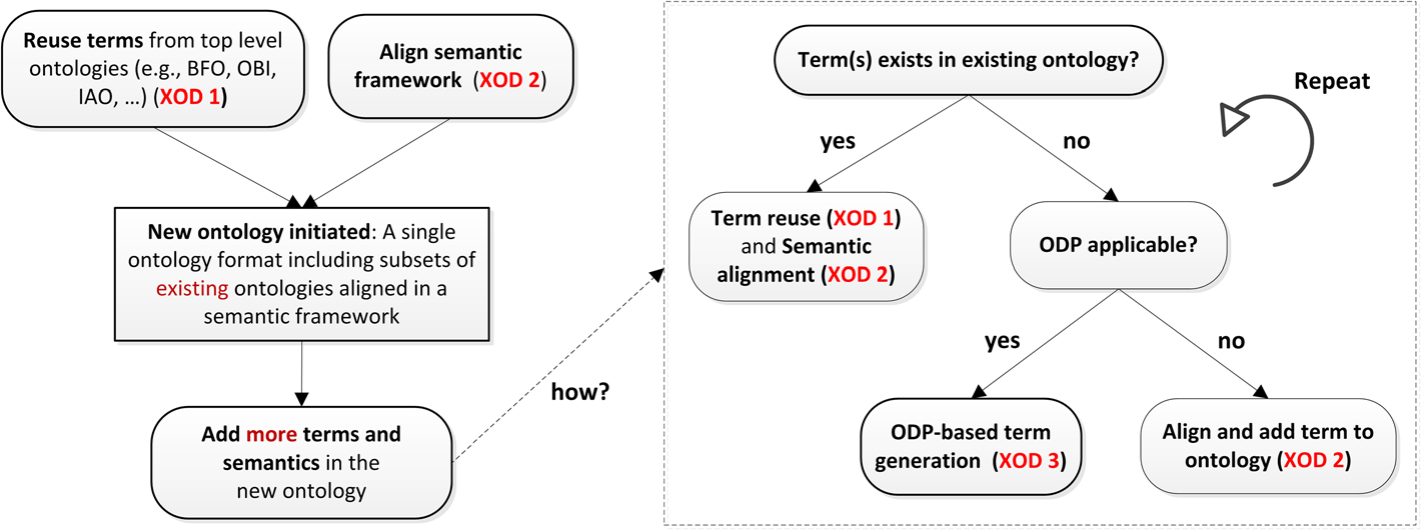
A general ontology development pipeline using XOD principles. To initiate a new ontology, needed terms from existing ontologies are imported and reused (XOD 1) and aligned together with other ontology terms in a consistent semantic framework (XOD 2). To add more terms and semantics afterwards, we can use the same XOD 1/2 methods to add terms from existing ontologies, and for new terms, we can either use ODP-based term generation strategy (XOD 3) and manually align and add terms to the new ontology. Community extensibility (XOD 4) should be considered and applied during the whole ontology development pipeline

The Ontorat program (http://ontorat.hegroup.org) supports ODP-based creation of new ontology terms, annotations and logical axioms [20]. Fig. 3 illustrates an example of using Ontorat to add new terms, annotations, and axioms to the Ontology of Adverse Events (OAE) [40]. Ontorat uses reusable ODPs (Fig. 4a) to automatically generate and edit ontology terms and axioms and provides term annotations. A specific ODP can be used to derive an Excel template of different terms/annotations and a set of rules that define the relations among those terms/annotations (Fig. 3b). The Ontorat template, similar to a QTT (Quick Term Template) originated by OBI developers [41], can be populated with specific terms or annotations to define or annotate specific ontology terms, or generate axioms illustrating logic relations between ontology terms. With the support of the Ontorat settings (Fig. 3c), the populated template spreadsheet can then be converted into an OWL file with newly generated ontology terms and axioms (Fig. 3d and e). The setting and template files can also be saved and reused.

Other ODP-based XOD tools include MappingMaster [42], TermGenie [43], Populous [44], Webulous [45], and ROBOT [36] (Table 1). Developed as a Protégé plugin, MappingMaster can only be used with old version Protégé 3.4 and is not available for newer Protégé 4 and 5 [42]. Targeting domain experts, TermGenie provides a web application that supports new GO term generation based on predefined patterns [43]. Populous requires software installation but provides a user-friendly interface [44]. Webulous is Google Add-On application usable with Google Spreadsheets [45]. ROBOT also has a template system for converting spreadsheets of terms to OWL files.

### XOD 4: Community extensibility

The community’s involvement during the developing phase of an ontology is the key for wide adoption of the ontology in the future. However, this step is often a bottleneck for ontology development, since the wider the community is, the more difficult it is to reach agreements on term definitions and classifications. In the reality, an ontology is often initiated by a small group and often driven by one or more use cases. To enhance its quality and broad recognition, XOD 4 recommends that a broader community with more developers and users participate in the ontology development and applications. This XOD community extensibility principle emphasizes the community participation to further extend, develop, and apply an ontology. With the principle of community extensibility, one ontology can be extended to cover different use cases in the same project and different projects from a wide range of research communities. The nature of such a practice will require more people to participate, make the ontology community bigger, and achieve better data interoperability.

WebProtege [46] and Ontokiwi [24] support community-based ontology development. WebProtege is a web-based ontology editor that supports collaborative OWL ontology development [46]. WebProtege has been used by many groups. It includes full change tracking and revision history, and many community collaboration features such as sharing and permissions, threaded notes and discussions, watches and email notifications. Ontokiwi is the user-friendly Wiki-like web program that supports community-wide ontology editing, annotation, discussion, and distribution [24]. Ontobedia is an Ontokiwi application preloaded with existing biomedical ontologies [24]. The Wiki-like addition and editing of text that is not part of the ontology makes Ontokiwi/Ontobedia a unique platform for community-wide ontology discussion and distribution.

For community-wide ontology development and applications, tools to support ontology query, comparison, and evaluations are also needed. NCBO BioPortal [2], OLS [47], Ontobee [21], and AberOWL [48] are commonly used ontology registry and repositories that also provide ontology visualization, queries and analysis features, which facilitates the community involvement principle. The SPOT ontology toolkit (http://www.ebi.ac.uk/spot/ontology/) also provides a list of community-driven open source ontology tools. For example, Ontobee (http://www.ontobee.org) is an ontology browser and a linked ontology data server for dereferencing ontology terms [21]. Ontobeep is an ontology comparison program that compares ontologies and identifies common terms existing in two or three ontologies by aligning 2–3 ontologies from the roots of these ontologies [22]. Ontobeep also detects inconsistency and term duplication in one or more ontologies.

#### Demonstrations of XOD implementation for interoperable ontology development

Figure 4 outlines a simple pipeline of how the XOD principles can be used together for productive ontology development. Basically, a new ontology can be initiated by reusing existing terms from different ontologies (XOD 1) and aligning these terms in a semantic framework (XOD 2), and new terms can be added by extending the semantic framework (XOD 2) and if ODPs identifiable, applying ODP-based approach (XOD 3). Ontology development often uses top-down and bottom-up approaches simultaneously [49]. The ontology initiation step is usually achieved by the top-down approach, i.e., developing the top level semantic framework by reusing and aligning upper level terms and semantics from existing ontologies (XOD 1/2). The same top-down approach can also be used to generate the upper level new terms commonly identified in the new ontology. Meanwhile, the bottom-up approach is use case driven and focuses on adding new terms to address specific use cases. For the bottom-up approach, XOD 1–3 principles are all important, and if possible, ODP-based design and term generation (XOD 3) is often critical to ensure development efficiency and consistency.

Here we will demonstrate our pipeline by using the complete use case of developing the community-based Vaccine Ontology (VO) [10, 50, 51]. As outlined in the extensive ontology development pipeline (Fig. 4), at the early stage of the VO development, we performed ontology survey and reused terms from several existing ontologies including BFO [33], OBI [7], GO [8], and the Information Artifact Ontology (IAO) [52]. The original VO version reported in 2009 included ~1000 imported terms from 10 existing ontologies and ~1000 VO-specific terms [50]. Since then more terms have been added to VO. As of November 20, 2017, out of 6541 terms in VO, approximately 1600 terms were imported and reused from approximately 30 ontologies (http://www.ontobee.org/ontostat/VO).

Many VO-specific terms were added to VO by semantically alignment with the upper BFO ontology or middle level ontologies (e.g., OBI) (XOD 2). For example, VO term ‘vaccine’ (VO_0000001) is asserted as a subclass of OBI term ‘processed material’ (OBI_0000047). This assertion means that any non-processed material (e.g., an infectious bacterium that exists in the air) that causes an infection in human and eventual immune responses and protection in the human is not counted as a vaccine. Similarly, the VO term ‘vaccination’ (VO_0000002) is asserted as a subclass of OBI term ‘administering substance in vivo’ (OBI_0600007). The alignment with ‘administering substance in vivo’ differentiates VO vaccination (i.e., administering a vaccine to in vivo) from immunization (i.e., to make one immune to something). In comparison, vaccination is considered as the synonym of immunization in MedDRA, a controlled terminology system commonly used for representation of regulatory activities [53].

In many cases, we can generate a number of new terms simultaneously by developing and following specific ODPs (XOD 3). For example, the VO developers retrieved from the US Department of Agriculture (USDA) and other public databases the information of approximately 800 licensed animal vaccines. Manually adding these animal vaccines to VO would be time consuming. To speed up the inclusion of the large number of licensed animal vaccines to VO, an ODP was developed to include different entities (e.g., vaccine name, manufacturer, animal species, animal pathogen, and disease), annotations, and the semantic relations among these entities. Such an ODP was further used to design an Excel template which was then applied to include the categorized information of these vaccines. Ontorat was finally used to automatically transfer the ODP and the information recorded in the Excel file to an OWL file and then imported to VO [20]. Furthermore, the same ODP could be used later to add new animal vaccines to VO. The Ontorat use case of ODP-based VO addition of veterinary vaccines was first presented in the 2012 International Conference for Biomedical Ontology (ICBO) [54]. Since then other ODPs were also developed for further VO development [51]. Meanwhile, it is noted that not all new terms can be fit under identifiable common design patterns. In this case, we can generate the term by aligning it with existing framework (XOD 2) (Fig. 4).

As a community-based open source ontology, the VO development has involved the broader community in its continuous development (XOD 4). The community participation helps further extend the VO and its interoperability with other biomedical ontologies. For example, according to BioPortal and Ontobee, the VO term vaccine (VO_0000001) has been reused by more than ten other ontologies such as OBI and Apollo Structured Vocabulary (https://github.com/apollodev/), and the VO term vaccination (VO_0000002) has been reused by ten other ontologies such as the Prescription of Drugs Ontology (https://github.com/OpenLHS/PDRO). In addition, the VO community involvement makes it achieve better data interoperability with other ontologies. Meanwhile, the community involvement extends the applications of VO, such as vaccine-related T cell and B cell response analysis and queries [55], epitope data management [56], vaccine-related literature mining [10], and vaccine-related network analysis [57, 58].

In addition to VO, many other ontologies, e.g., Beta Cell Genomics Ontology (BCGO) [37], MicrO ontology for representing microorganism phenotypic and metabolic characters [59], and BioAssay Ontology (BAO) [60], have been developed using the same or similar strategies.

## Discussion and perspectives

The XOD strategy and principles reflect the growing maturity of biological and biomedical ontology development. When only a small number of ontologies were developed, such XOD strategy was not needed. However, with hundreds of ontologies developed now, it is critical to ensure ontology interoperability, and the XOD principles provide a practical solution. Given the importance of ontologies in the integration, sharing, and analysis of the increasing large and heterogeneous data/metadata and knowledge, the XOD strategy is very significant and critical to meet the challenges in the current big data era.

Among the four XOD principles, the first three principles emphasize the requirements to reuse ontology terms, extend and align semantic structures, and build new terms and semantics among terms using design patterns. XOD 2 is a more general principle which covers the semantic interoperability among terms including terms from the target ontology and terms newly generated or imported from source ontology. Extending XOD 1 and 2, XOD 3 provides a more specific mechanism (i.e., ODP-based term generation and editing) to achieve consistent ontology term generation and annotation. While the first three principles provide more technical guidance, XOD 4 emphasizes the community collaboration and involvement in new ontology development.

XOD is complementary to the OBO principles [1] and the ten simple rules proposed for ontology development [26]. The OBO principles (e.g., open, common format, versioning, scope, relations, users, collaboration, and locus of authority) provide general principles for the development of an ontology (http://obofoundry.org/principles/fp-000-summary.html). The ten simple rules proposed by Malone et al. include ontology term reuse, design patterns, and community engagement [26], which are directly associated with XOD principles. The other 7 rules (e.g., scope, license, versioning) are not directly related. In comparison to the OBO principles and the ten simple rules, the XOD principles address the single important point of ontology extensibility and emphasize different scales of extensible relations among ontologies, with the aim to achieve ontology interoperability. Since different ontologies extend and are aligned with existing reliable ontologies, applying the XOD principles will support the OBO aim of establishing non-redundant and interoperable suite of ontologies.

The XOD strategy supports the FAIR Guiding Principles, which propose that various data be Findable, Accessible, Interoperable and Reusable [27]. Ontologies lay out the basic foundation for the data FAIRness. Adopting the XOD strategy will lead to the development of extensible ontologies and the generation of ontology-extended data and metadata representations. Such ontology-supported data sharing and integration will result in natural data access, interoperability, and usability, query, and advanced analysis. For example, the KaBOB knowledge base uses the OBO ontologies to semantically integrate data from 18 prominent biomedical databases [61]. Millions of RDF triples were also generated in KaBOB, enabling findable, accessible, interoperable, and reusable queries of the underlying data from these databases. Therefore, the XOD strategy supports the eventual achievement of the FAIRness of data.

Many challenges exist in adopting and achieving the goals defined in the XOD strategy and principles. First, the interoperability among current hundreds of ontologies is still limited and challenging [32, 62, 63]. Term redundancy among ontologies cannot be solved easily, leading to issues of achieving data FAIRness. Second, only a small amount of data resources (including a large number of databases) adopt ontology-guided strategy, which restricts data interoperability and analysis. Third, while many linked data systems [25] standardize data using ontologies, the ontologies underlying linked data are often non-interoperable, making linked data systems become individual silos and difficult to integrate [64, 65]. To address these challenges, it is important to adopt the XOD strategy and XOD principles. Active ontology training and outreach will be beneficial.

The suite of Ontoanimal tools has provided different features to address several real issues in ontology development. Each of these tools focuses on one or more primary tasks, and all together they are combined to strongly support XOD principles. Given the complexity of these tools, there are concerns about their usability and sustainability. Since these tools are more about research in ontology development, it is important to have a strong evaluation system to be used to better understand the strengths and limitation of each tool.

While Ontoanimal tools and other similar XOD tools have already supported the XOD strategy, existing tools require further improvements, and more user-friendly integrative tools are needed. Tools are critical to make more efficient extensible ontology development. For example, although it was recognized that term reuse was a better strategy, the term reuse principle was not widely implemented until Ontofox and other tools were developed. Currently XOD tool usage often requires extensive training. More easy-to-use and integrative XOD tools are desired for ontology developers and users with no or limited programming background. We believe that the adoption of the XOD principles together with robust XOD tools would greatly support interoperable ontology development and data FAIRness.

## Conclusion

Our examination of Ontoanimal tools and similar programs discovered their shared features of extensibility. We proposed the “eXtensible Ontology Development” (XOD) strategy and four XOD principles to support extensible ontology development and usage. We propose to adopt these XOD principles for active development and usage of extensible ontologies and tools, leading to better data FAIRness.

## Abbreviations

BFO: Basic formal ontology
GO: Gene ontology
MIREOT: Minimum Information to reference an external ontology term
NCBO: National Center for Biomedical Ontology
OAE: Ontology of adverse events
OBO: Open biological and biomedical ontologies
ODP: Ontology design pattern
OWL: Web ontology language
RDF: Resource description framework
XML: eXtensible markup language
XOD: eXtensible ontology development
